# Evaluating the effectiveness of an artificial intelligence–based chatbot for reducing dental anxiety among adult dental patients

**DOI:** 10.3389/froh.2026.1778200

**Published:** 2026-04-13

**Authors:** Ahmed A. Madfa, Abdullah F. Alshammari, Bassam A. Alanazi, Sulaiman N. Alshammari

**Affiliations:** 1 Department of Restorative Dental Science, College of Dentistry, University of Ha’il, Ha’il, Saudi Arabia; 2 Department of Basic Dental and Medical Science, College of Dentistry, University of Ha’il, Ha’il, Saudi Arabia; 3 Dental Research Centre, College of Dentistry, University of Ha’il, Ha’il, Saudi Arabia; 4 Dental Student, College of Dentistry, University of Ha’il, Ha’il, Saudi Arabia

**Keywords:** anxiety management, artificial intelligence, chatbot, dental anxiety, digital health, patient education

## Abstract

**Background:**

Dental anxiety is a common psychological barrier that negatively influences dental attendance, patient cooperation, and treatment outcomes. Traditional anxiety-management strategies are often resource-intensive and difficult to implement routinely. Artificial intelligence (AI)–based chatbots offer a scalable and accessible digital approach that may help reduce dental anxiety through patient education, reassurance, and emotional support.

**Methods:**

A quasi-experimental pre–post interventional study was conducted among 415 adult dental patients attending the outpatient clinics of the University of Ha'il, Saudi Arabia. Participants interacted with an AI-based chatbot for 10–15 min prior to treatment. Dental anxiety was assessed before and immediately after chatbot use using the Modified Dental Anxiety Scale (MDAS) across five common dental scenarios. Demographic variables, including gender, age, educational level, and monthly income, were analysed. Chi-square tests and Spearman's correlation were applied to evaluate changes in the severity of dental anxiety and associations between pre- and post-intervention scores.

**Results:**

A statistically significant reduction in dental anxiety was observed across all five dental scenarios following chatbot interaction (*p* < 0.001). The greatest reductions were noted for invasive procedures, particularly tooth drilling and local anaesthetic injection. The severity of dental anxiety decreased consistently across gender, age, educational, and income groups, indicating a broadly similar effect of the intervention across these subgroups. Although patients with greater baseline severity of dental anxiety remained relatively more anxious post-intervention, the overall severity of anticipatory anxiety was substantially reduced. Female patients exhibited higher baseline anxiety, but gender-based differences were markedly reduced after chatbot use.

**Conclusion:**

The use of the AI-based chatbot demonstrated a consistent association with reduced anticipatory dental anxiety across diverse patient groups and clinical scenarios. As a low-cost, scalable, and patient-centred tool, chatbot-based support may complement conventional anxiety-management strategies and support the patient experience in routine dental care.

## Introduction

Dental anxiety refers to a persistent and excessive fear or apprehension related to specific dental situations or stimuli, characterised by marked anxiety or panic responses when anticipating or encountering dental procedures. Consistent with DSM-V criteria for specific phobia, dental anxiety involves disproportionate fear relative to the actual threat, active avoidance of dental care, and clinically significant distress or impairment in functioning. This anxiety may be triggered by cues such as dental instruments, injections, clinical sounds, or the dental environment, and is maintained by previous negative experiences, conditioned learning, and maladaptive cognitive appraisals (1–6).

Dental anxiety is increasingly recognised as a complex and multidimensional construct that encompasses emotional, cognitive, behavioural, and physiological components rather than a single, uniform response to dental care. It reflects an interaction between past experiences, individual temperament, perceived control, pain expectancy, sociocultural influences, and contextual features of the dental environment. Consequently, dental anxiety manifests heterogeneously across individuals and clinical situations, which has important implications for how interventions are conceptualised, evaluated, and compared (5, 6).

Background factors related to dental anxiety involve a combination of previous negative dental experiences, fear of pain, perceived loss of control, limited understanding of dental procedures, and broader psychological and contextual influences (7–9). Patients frequently report that certain dental procedures, such as sitting in the reception area, hearing or seeing dental drills, receiving local anaesthetic injections, scaling and polishing, and anticipating a dental appointment, cause them a great deal of anxiety (10–12). These procedure-specific triggers stand for crucial points in the dental treatment pathway where anxiety may peak and affect patient behaviour.

Dental anxiety affects patients across all age groups, although its frequency and severity vary according to socioeconomic and demographic factors. Previous research has shown that younger individuals, female patients, and those with lower income or educational attainment are more likely to experience severe dental anxiety (13–17). These discrepancies highlight the necessity of customised, patient-centred strategies—defined here as approaches that prioritise patients' informational needs, emotional comfort, and perceived control through clear, procedure-specific explanations—and underscore the importance of considering individual patient characteristics when assessing anxiety-reducing interventions.

Conventional approaches to dental anxiety management include behavioural techniques, enhanced dentist–patient communication, psychological interventions (e.g., cognitive behavioural therapy), and pharmacological sedation. These strategies primarily target established or chronic dental anxiety and are typically delivered within or alongside clinical treatment. While effective for selected patients, they often require additional time, specialist expertise, and clinical resources, limiting their routine applicability for managing pre-appointment or anticipatory anxiety in busy dental settings. This has motivated interest in complementary, low-intensity digital tools that can be deployed prior to treatment to address situational anxiety rather than replace established therapeutic approaches (18, 19).

Chatbots offer interactive, tailored, and on-demand information using natural language processing, enabling patients to ask questions, get clarification on concerns, and feel reassured in a nonjudgmental setting (20–22). Prior research has investigated AI-based tools for providing dental and health information (23, 24), but these applications mostly provided static question-answer forms or were topic-specific (e.g., dental implant inquiries) and lacked interactive, scenario-based assistance. The modern AI chatbot, on the other hand, offers interactive coping mechanisms, real-time emotional support, and patient-centered, scenario-specific teaching. This conversational method improves user involvement and may lessen anticipatory dental anxiety by enabling customized replies in a variety of dental scenarios.

By improving procedural comprehension, clarifying misunderstandings, providing brief supportive information, and enhancing patients' perceptions of control over their care, chatbots may reduce short-term anticipatory dental anxiety immediately prior to treatment, rather than treating dental anxiety in a clinical or long-term sense (9, 25, 26). Empirical research explicitly focusing on dental patients remains limited, despite evidence from mental health and general medical contexts supporting the potential of chatbot-based interventions in reducing anxiety.

Accordingly, the present study aimed to examine the immediate effect of an AI-based chatbot on anticipatory dental anxiety prior to treatment. Rather than evaluating a therapeutic intervention, the chatbot functioned as a brief, scenario-based informational and supportive tool delivered immediately before dental care. The study assessed changes in the severity of anticipatory dental anxiety across common dental situations and explored whether these changes differed by gender, age, educational level, and monthly income using a pre–post quasi-experimental design.

## Methods

The study was conducted at the outpatient dental clinics of the University of Ha'il, Saudi Arabia, from September 8 to December 12, 2025.

Ethical approval was obtained from the Institutional Review Board of the University of Ha'il. Before participating, participants gave written informed consent. In compliance with international ethical norms for research involving human subjects, participation was voluntary, anonymity was guaranteed, and all data obtained were anonymised and used exclusively for the study. Furthermore, the chatbot (Chatbase) was configured to be completely anonymous, and no personally identifiable information was collected or stored during the interactions to ensure the highest level of data privacy.

Participants were dental patients who visited the outpatient clinics during the research period. The requirements for inclusion were (1) 18 years of age or older; (2) attending any non-emergency dental treatment; and (3) being able to read and comprehend the questionnaire. Patients who had previously used comparable chatbot-based anxiety therapies, had recognised mental illness, had urgent tooth pain that needed to be treated immediately, or had insufficient questionnaire responses were omitted.

Participants were recruited consecutively in the clinic waiting rooms. Pre- and post-intervention evaluations were performed by 415 patients who satisfied the inclusion criteria.

G*Power version 3.1 was used to estimate the sample size for paired proportion comparisons. The minimum necessary sample size was determined to be 367 patients, assuming an effect size of 0.2, a significance level (*α*) of 0.05, and statistical power of 80%. The final sample of 415 patients had sufficient power for subgroup analysis, after the intended sample size was adjusted by 15%–20% to account for possible dropouts or incomplete responses.

The AI-based chatbot was created utilizing the Chatbase platform and adhered to evidence-based recommendations for treating dental anxiety (9, 25), as per Chi's assessment (27). Accurate information on 17 dental anxiety measures, such as Dental Anxiety Scale (DAS), The Modified Dental Anxiety Scale (MDAS), and Dental Fear Survey (DFS), was made possible by its knowledge base, which was taken from Chi (27). The AI was limited to this source by a stringent system prompt, guaranteeing dependability and avoiding data that was beyond the purview of the study. Five dental situations that cause anxiety were addressed by the chatbot: scheduling an appointment, waiting in the clinic, drilling teeth, scaling and cleaning teeth, and administering local anesthetic (10–12, 27). It supplied organized, interactive advice, explained processes, cleared up misunderstandings, recommended coping mechanisms, and provided emotional support for individuals.

It was created to address five dental scenarios that are frequently linked to increased patient anxiety: preparing for a dental appointment, waiting in the dentist's office, drilling teeth, scaling and cleaning teeth, and giving local anaesthetic injections. The chatbot provided organised, interactive teaching information for each of these situations, including pertinent processes, addressing common misconceptions and concerns, offering coping mechanisms, and providing emotional support. Before receiving dental treatment, patients used their own mobile phones or clinic-provided devices to communicate with the chatbot for 10–15 min.

Data collection was conducted in three sequential phases. First, participants completed an electronic pre-intervention questionnaire assessing the baseline severity of dental anxiety. Second, participants interacted with the AI-based chatbot. Third, they completed a post-intervention questionnaire immediately after the interaction and prior to receiving dental treatment to reassess the severity of anticipatory dental anxiety. In this process, each participant completed all five scenarios to ensure a consistent and comprehensive assessment across all evaluated clinical situations. To facilitate paired analysis while preserving anonymity, each participant's responses were coded.

MDAS, a validated 5-question tool, was used to measure dental anxiety. Responses were rated on a 5-point Likert scale from 1 (not nervous) to 5 (very anxious). Greater dental anxiety is indicated by higher total scores, which range from 5 to 25. In dental patient populations, the MDAS has shown excellent validity and reliability. The MDAS has demonstrated excellent reliability and construct validity in dental populations (28, 29). A total MDAS score of ≥19 is the validated cut-off for severe dental anxiety.

Patient demographic variables included monthly income, gender, age group, and level of education. These variables were analysed to explore whether the effect of the chatbot differed across socioeconomic and demographic groups and to examine variations in the baseline severity of dental anxiety.

Statistical analysis was performed using SPSS version 26.0 (IBM Corp., Armonk, NY, USA). Descriptive statistics (frequencies and percentages) were calculated for demographic characteristics and the severity of dental anxiety. Chi-square tests were used to compare the categorical distribution of dental anxiety severity across all MDAS items and patient subgroups before and after chatbot use. Spearman's rank correlation coefficient was applied to assess associations between pre- and post-intervention MDAS scores. Statistical significance was defined as a *p*-value of less than 0.05.

## Results

### Participant characteristics

A total of 415 patients completed both the pre- and post-intervention assessments and were included in the final analysis. Participants represented a wide range of age groups, educational levels, and monthly income categories, with both male and female patients included. Dental anxiety was assessed across five common dental scenarios using the MDAS before and immediately after interaction with the AI-based chatbot.

### Changes in dental anxiety before and after chatbot interaction

Table 1 shows the distribution of dental anxiety severity across the five MDAS components both before and after using a chatbot. After the session, chi-square analysis showed a statistically significant change in the categorical distribution of anxiety levels for each of the five dental situations (*p* < 0.001). In particular, following chatbot participation, the percentage of participants who reported “not anxious” or “slightly anxious” responses increased, but the percentage who reported “very anxious” or “extremely anxious” responses fell across all items.

**Table 1:** Distribution of the severity of dental anxiety before and after Use of the AI-based chatbot across different dental situations.

MDAS Item	Time	Not anxious *n* (%)	Slightly anxious *n* (%)	Moderately anxious *n* (%)	Very anxious *n* (%)	Extremely anxious *n* (%)	*p*-value
Q1. If you were to go to the dentist tomorrow for treatment, how would you feel?	Before	154 (37.1)	109 (26.3)	99 (23.9)	40 (9.6)	13 (3.1)	<.001
	After	236 (56.9)	116 (28.0)	43 (10.4)	12 (2.9)	8 (1.9)	
Q2. If you were sitting in the waiting room (waiting for treatment), how would you feel?	Before	114 (27.5)	139 (33.5)	96 (23.1)	51 (12.3)	15 (3.6)	<.001
	After	228 (54.9)	123 (29.6)	47 (11.3)	12 (2.9)	5 (1.2)	
Q3. If you were about to have one of your teeth drilled (using the drill), how would you feel?	Before	54 (13.0)	124 (29.9)	96 (23.1)	87 (21.0)	54 (13.0)	<.001
	After	169 (40.7)	149 (35.9)	58 (14.0)	17 (4.1)	22 (5.3)	
Q4. If you were about to have your teeth cleaned and polished (removal of tartar and stains), how would you feel?	Before	107 (25.8)	156 (37.6)	72 (17.3)	57 (13.7)	23 (5.5)	<.001
	After	226 (54.5)	125 (30.1)	45 (10.8)	11 (2.7)	8 (1.9)	
Q5. If you were about to receive a local anesthetic injection in the gum above one of the upper back teeth, how would you feel?	Before	56 (13.5)	85 (20.5)	88 (21.2)	79 (19.0)	107 (25.8)	<.001
	After	187 (45.1)	137 (33.0)	43 (10.4)	26 (6.3)	22 (5.3)	

The largest changes in anxiety distribution were observed for invasive procedures, particularly tooth drilling (Q3) and local anaesthetic injection (Q5), which showed the highest baseline levels of moderate-to-severe anxiety. These situations showed significant decreases in higher anxiety categories after the intervention, suggesting a significant change toward less anticipatory worry right before treatment.

### Gender-Based differences

Female participants reported higher baseline dental anxiety than men for a number of situations, as seen in Table 2. Differences were statistically significant for waiting in the dentist office (Q2), anticipating a dental visit (Q1), and having their teeth drilled (Q3). Chi-square comparisons showed substantial decreases in anxiety severity for both genders in all five scenarios following chatbot use (*p* < 0.001). Gender disparities were significantly lessened after the intervention, indicating that both groups were moving toward lower anxiety categories.

**Table 2:** Comparison of the severity of dental anxiety between genders before and after AI-based chatbot intervention.

Q No	Gender	Before using the chatbot						After using the chatbot					
		A *n* (%)	B *n* (%)	C *n* (%)	D *n* (%)	E *n* (%)	*p*-value	A *n* (%)	B *n* (%)	C *n* (%)	D *n* (%)	E *n* (%)	*p*-value
Q1	Male	130 (47.3)	58 (21.1)	62 (22.5)	19 (6.9)	6 (2.2)	<.001	179 (65.1)	66 (24.0)	22 (8.0)	6 (2.2)	2 (0.7)	<.001
	Female	24 (17.1)	51 (36.4)	37 (26.4)	21 (15.0)	7 (5.0)		57 (40.7)	50 (35.7)	21 (15.0)	6 (4.3)	6 (4.3)	
Q2	Male	85 (30.9)	100 (36.4)	59 (21.5)	24 (8.7)	7 (2.5)	.002	173 (62.9)	71 (25.8)	26 (9.5)	4 (1.5)	1 (0.4)	<.001
	Female	29 (20.7)	39 (27.9)	37 (26.4)	27 (19.3)	8 (5.7)		55 (39.3)	52 (37.1)	21 (15.0)	8 (5.7)	4 (2.9)	
Q3	Male	42 (15.3)	89 (32.4)	60 (21.8)	57 (20.7)	27 (9.8)	.018	130 (47.3)	101 (36.7)	28 (10.2)	10 (3.6)	6 (2.2)	<.001
	Female	12 (8.6)	35 (25.0)	36 (25.7)	30 (21.4)	27 (19.3)		39 (27.9)	48 (34.3)	30 (21.4)	7 (5.0)	16 (11.4)	
Q4	Male	72 (26.2)	110 (40.0)	43 (15.6)	38 (13.8)	12 (4.4)	.316	165 (60.0)	75 (27.3)	25 (9.1)	7 (2.5)	3 (1.1)	.017
	Female	35 (25.0)	46 (32.9)	29 (20.7)	19 (13.6)	11 (7.9)		61 (43.6)	50 (35.7)	20 (14.3)	4 (2.9)	5 (3.6)	
Q5	Male	39 (14.2)	53 (19.3)	57 (20.7)	46 (16.7)	80 (29.1)	.153	138 (50.2)	91 (33.1)	23 (8.4)	14 (5.1)	9 (3.3)	.003
	Female	17 (12.1)	32 (22.9)	31 (22.1)	33 (23.6)	27 (19.3)		49 (35.0)	46 (32.9)	20 (14.3)	12 (8.6)	13 (9.3)	

A, Not anxious; B, Slightly anxious; C, Moderately anxious; D, Very anxious; E, Extremely anxious.

### Educational level and dental anxiety

Participants with lower levels of education were more likely to experience moderate-to-severe anxiety, especially for procedures involving drilling and injections (Table 3), which showed that baseline anxiety intensity differed by educational level. All educational groups showed substantial movements toward lower anxiety categories for all MDAS items after interacting with the chatbot (*p* < 0.001). These results show that, irrespective of educational background, the chatbot intervention was linked to decreased anticipatory anxiety.

**Table 3:** Comparison of the severity of dental anxiety across educational levels before and after Use of the AI-based chatbot.

Q No	Educational level	Before using the chatbot						After using the chatbot					
		A *n* (%)	B *n* (%)	C *n* (%)	D *n* (%)	E *n* (%)	*p*-value	A *n* (%)	B *n* (%)	C *n* (%)	D *n* (%)	E *n* (%)	*p*-value
Q1	Nonformal/ General	20 (37.7)	15 (28.3)	12 (22.6)	4 (7.5)	2 (3.8)	**<**.**001**	33 (62.3)	14 (26.4)	4 (7.5)	1 (1.9)	1 (1.9)	**<**.**001**
	Diploma	29 (29.9)	31 (32.0)	26 (26.8)	8 (8.2)	3 (3.1)		53 (54.6)	31 (32.0)	9 (9.3)	2 (2.1)	2 (2.1)	
	Bachelor	78 (35.9)	57 (26.3)	56 (25.8)	20 (9.2)	6 (2.8)		123 (56.7)	58 (26.7)	27 (12.4)	6 (2.8)	3 (1.4)	
	Postgraduate	27 (57.4)	6 (12.8)	5 (10.6)	8 (17.0)	1 (2.1)		27 (57.4)	13 (27.7)	3 (6.4)	3 (6.4)	1 (2.1)	
Q2	Nonformal/ General	16 (30.2)	18 (34.0)	11 (20.8)	6 (11.3)	2 (3.8)	**<**.**001**	31 (58.5)	15 (28.3)	5 (9.4)	1 (1.9)	1 (1.9)	**<**.**001**
	Diploma	21 (21.6)	36 (37.1)	26 (26.8)	10 (10.3)	4 (4.1)		51 (52.6)	32 (33.0)	10 (10.3)	2 (2.1)	2 (2.1)	
	Bachelor	55 (25.3)	72 (33.2)	55 (25.3)	28 (12.9)	7 (3.2)		118 (54.4)	64 (29.5)	26 (12.0)	7 (3.2)	2 (0.9)	
	Postgraduate	22 (46.8)	13 (27.7)	4 (8.5)	7 (14.9)	1 (2.1)		10 (21.3)	12 (25.5)	6 (12.8)	2 (4.3)	17 (36.2)	
Q3	Nonformal/ General	7 (13.2)	17 (32.1)	12 (22.6)	11 (20.8)	6 (11.3)	**<**.**001**	21 (39.6)	19 (35.8)	8 (15.1)	2 (3.8)	3 (5.7)	**<**.**001**
	Diploma	11 (11.3)	31 (32.0)	21 (21.6)	23 (23.7)	11 (11.3)		39 (40.2)	37 (38.1)	13 (13.4)	4 (4.1)	4 (4.1)	
	Bachelor	26 (12.0)	63 (29.0)	52 (24.0)	45 (20.7)	31 (14.3)		91 (41.9)	76 (35.0)	31 (14.3)	9 (4.1)	10 (4.6)	
	Postgraduate	10 (21.3)	13 (27.7)	11 (23.4)	8 (17.0)	5 (10.6)		18 (38.3)	17 (36.2)	6 (12.8)	2 (4.3)	4 (8.5)	
Q4	Nonformal/ General	14 (26.4)	21 (39.6)	8 (15.1)	6 (11.3)	4 (7.5)	.**001**	29 (54.7)	17 (32.1)	5 (9.4)	1 (1.9)	1 (1.9)	**<**.**001**
	Diploma	26 (26.8)	38 (39.2)	14 (14.4)	13 (13.4)	6 (6.2)		52 (53.6)	31 (32.0)	9 (9.3)	3 (3.1)	2 (2.1)	
	Bachelor	56 (25.8)	83 (38.2)	39 (18.0)	30 (13.8)	9 (4.1)		122 (56.2)	62 (28.6)	23 (10.6)	6 (2.8)	4 (1.8)	
	Postgraduate	11 (23.4)	14 (29.8)	11 (23.4)	8 (17.0)	3 (6.4)		23 (48.9)	15 (31.9)	8 (17.0)	1 (2.1)	0 (0.0)	
Q5	Nonformal/ General	6 (11.3)	11 (20.8)	10 (18.9)	8 (15.1)	18 (34.0)	**<**.**001**	25 (47.2)	17 (32.1)	5 (9.4)	3 (5.7)	3 (5.7)	**<**.**001**
	Diploma	11 (11.3)	19 (19.6)	20 (20.6)	18 (18.6)	29 (29.9)		44 (45.4)	33 (34.0)	9 (9.3)	5 (5.2)	6 (6.2)	
	Bachelor	30 (13.8)	45 (20.7)	48 (22.1)	43 (19.8)	51 (23.5)		98 (45.2)	70 (32.3)	22 (10.1)	14 (6.5)	13 (6.0)	
	Postgraduate	9 (19.1)	10 (21.3)	10 (21.3)	10 (21.3)	8 (17.0)		20 (42.6)	17 (36.2)	7 (14.9)	4 (8.5)	0 (0.0)	

Bold values indicate statistically significant differences (*p* < 0.05). A, Not anxious; B, Slightly anxious; C, Moderately anxious; D, Very anxious; E, Extremely anxious. *p*-values represent comparisons across groups using the chi-square test.

### Income and dental anxiety

All income groups reported moderate-to-severe dental anxiety at baseline, particularly for invasive treatments (Table 4). Chi-square tests conducted after the intervention revealed statistically significant decreases in the level of anxiety in every dental scenario and in every income category (*p* < 0.001). It appears that the observed decrease in anxiety was independent of socioeconomic position because the distributional movements toward lower anxiety categories were similar across income levels.

**Table 4:** Comparison of the severity of dental anxiety across income groups before and after Use of the AI-based chatbot.

Q No	Income level	Before using the chatbot						After using the chatbot					
		A *n* (%)	B *n* (%)	C *n* (%)	D *n* (%)	E *n* (%)	*p*-value	A *n* (%)	B *n* (%)	C *n* (%)	D *n* (%)	E *n* (%)	*p*-value
Q1	<4,000	44 (46.3)	23 (24.2)	20 (21.1)	6 (6.3)	2 (2.1)	**<**.**001**	53 (55.8)	27 (28.4)	10 (10.5)	3 (3.2)	2 (2.1)	**<**.**001**
	4,000–8,000	22 (31.9)	21 (30.4)	17 (24.6)	7 (10.1)	2 (2.9)		37 (53.6)	20 (29.0)	8 (11.6)	3 (4.3)	1 (1.4)	
	8,000–12,000	33 (29.7)	29 (26.1)	31 (27.9)	13 (11.7)	5 (4.5)		65 (58.6)	30 (27.0)	11 (9.9)	3 (2.7)	2 (1.8)	
	12,000–15,000	29 (42.6)	19 (27.9)	12 (17.6)	6 (8.8)	2 (2.9)		40 (58.8)	19 (27.9)	6 (8.8)	2 (2.9)	1 (1.5)	
	>15,000	26 (36.1)	17 (23.6)	19 (26.4)	8 (11.1)	2 (2.8)		41 (56.9)	20 (27.8)	8 (11.1)	1 (1.4)	2 (2.8)	
Q2	<4,000	29 (30.5)	34 (35.8)	21 (22.1)	8 (8.4)	3 (3.2)	**<**.**001**	**52** (**54.7)**	**28** (**29.5)**	**10** (**10.5)**	**3** (**3.2)**	**2** (**2.1)**	**<**.**001**
	4,000–8,000	17 (24.6)	24 (34.8)	16 (23.2)	8 (11.6)	4 (5.8)		**37** (**53.6)**	**21** (**30.4)**	**8** (**11.6)**	**2** (**2.9)**	**1** (**1.4)**	
	8,000–12,000	29 (26.1)	39 (35.1)	26 (23.4)	13 (11.7)	4 (3.6)		**62** (**55.9)**	**33** (**29.7)**	**12** (**10.8)**	**3** (**2.7)**	**1** (**0.9)**	
	12,000–15,000	20 (29.4)	20 (29.4)	17 (25.0)	9 (13.2)	2 (2.9)		**38** (**55.9)**	**21** (**30.9)**	**6** (**8.8)**	**2** (**2.9)**	**1** (**1.5)**	
	>15,000	19 (26.4)	22 (30.6)	16 (22.2)	13 (18.1)	2 (2.8)		**39** (**54.2)**	**20** (**27.8)**	**11** (**15.3)**	**2** (**2.8)**	**0** (**0.0)**	
Q3	<4,000	10 (10.5)	28 (29.5)	22 (23.2)	21 (22.1)	14 (14.7)	**<**.**001**	**38** (**40.0)**	**36** (**37.9)**	**13** (**13.7)**	**4** (**4.2)**	**4** (**4.2)**	**<**.**001**
	4,000–8,000	6 (8.7)	19 (27.5)	17 (24.6)	15 (21.7)	12 (17.4)		**28** (**40.6)**	**25** (**36.2)**	**10** (**14.5)**	**3** (**4.3)**	**3** (**4.3)**	
	8,000–12,000	16 (14.4)	34 (30.6)	25 (22.5)	24 (21.6)	12 (10.8)		**46** (**41.4)**	**38** (**34.2)**	**17** (**15.3)**	**5** (**4.5)**	**5** (**4.5)**	
	12,000–15,000	10 (14.7)	20 (29.4)	18 (26.5)	11 (16.2)	9 (13.2)		**27** (**39.7)**	**26** (**38.2)**	**9** (**13.2)**	**3** (**4.4)**	**3** (**4.4)**	
	>15,000	12 (16.7)	23 (31.9)	14 (19.4)	16 (22.2)	7 (9.7)		**30** (**41.7)**	**24** (**33.3)**	**9** (**12.5)**	**2** (**2.8)**	**7** (**9.7)**	
Q4	<4,000	23 (24.2)	39 (41.1)	14 (14.7)	12 (12.6)	7 (7.4)	.**001**	52 (54.7)	29 (30.5)	9 (9.5)	3 (3.2)	2 (2.1)	**<**.**001**
	4,000–8,000	19 (27.5)	24 (34.8)	13 (18.8)	9 (13.0)	4 (5.8)		37 (53.6)	21 (30.4)	8 (11.6)	2 (2.9)	1 (1.4)	
	8,000–12,000	27 (24.3)	44 (39.6)	21 (18.9)	13 (11.7)	6 (5.4)		61 (55.0)	33 (29.7)	12 (10.8)	3 (2.7)	2 (1.8)	
	12,000–15,000	19 (27.9)	24 (35.3)	12 (17.6)	9 (13.2)	4 (5.9)		37 (54.4)	21 (30.9)	7 (10.3)	2 (2.9)	1 (1.5)	
	>15,000	19 (26.4)	25 (34.7)	12 (16.7)	14 (19.4)	2 (2.8)		39 (54.2)	21 (29.2)	9 (12.5)	1 (1.4)	2 (2.8)	
Q5	<4,000	10 (10.5)	16 (16.8)	20 (21.1)	17 (17.9)	32 (33.7)	**<**.**001**	42 (44.2)	33 (34.7)	10 (10.5)	5 (5.3)	5 (5.3)	**<**.**001**
	4,000–8,000	8 (11.6)	14 (20.3)	14 (20.3)	13 (18.8)	20 (29.0)		31 (44.9)	23 (33.3)	8 (11.6)	3 (4.3)	4 (5.8)	
	8,000–12,000	16 (14.4)	22 (19.8)	23 (20.7)	22 (19.8)	28 (25.2)		51 (45.9)	36 (32.4)	11 (9.9)	6 (5.4)	7 (6.3)	
	12,000–15,000	12 (17.6)	17 (25.0)	14 (20.6)	11 (16.2)	14 (20.6)		30 (44.1)	23 (33.8)	7 (10.3)	4 (5.9)	4 (5.9)	
	>15,000	10 (13.9)	16 (22.2)	17 (23.6)	16 (22.2)	13 (18.1)		33 (45.8)	22 (30.6)	7 (9.7)	8 (11.1)	2 (2.8)	

Bold values indicate statistically significant differences (*p* < 0.05). A, Not anxious; B, Slightly anxious; C, Moderately anxious; D, Very anxious; E, Extremely anxious. *p*-values represent comparisons across groups using the chi-square test.

### Age-Based differences

Baseline dental anxiety varied by age group (Table 5), with younger and middle-aged individuals reporting higher levels of anxiety for injections and drilling. All age groups showed statistically significant decreases in anxiety intensity after using chatbots in all five situations (*p* < 0.001), with consistent changes toward lower anxiety categories, suggesting that chatbots are useful across the adult age range.

**Table 5:** Comparison of the severity of dental anxiety across different Age groups before and after using the AI-based chatbot.

Q No	Age group	Before using the chatbot						After using the chatbot					
		A *n* (%)	B *n* (%)	C *n* (%)	D *n* (%)	E *n* (%)	*p*-value	A *n* (%)	B *n* (%)	C *n* (%)	D *n* (%)	E *n* (%)	*p*-value
Q1	<18	12 (57.1)	4 (19.0)	4 (19.0)	1 (4.8)	0 (0.0)	**<**.**001**	14 (66.7)	5 (23.8)	1 (4.8)	0 (0.0)	1 (4.8)	**<**.**001**
	18–25	26 (33.8)	21 (27.3)	25 (32.5)	5 (6.5)	0 (0.0)		38 (49.4)	26 (33.8)	10 (13.0)	2 (2.6)	1 (1.3)	
	26–35	26 (23.6)	31 (28.2)	40 (36.4)	12 (10.9)	1 (0.9)		60 (54.5)	34 (30.9)	12 (10.9)	3 (2.7)	1 (0.9)	
	36–45	41 (35.0)	33 (28.2)	23 (19.7)	17 (14.5)	3 (2.6)		68 (58.1)	30 (25.6)	13 (11.1)	4 (3.4)	2 (1.7)	
	>45	49 (54.4)	20 (22.2)	7 (7.8)	5 (5.6)	9 (10.0)		56 (62.2)	21 (23.3)	7 (7.8)	3 (3.3)	3 (3.3)	
Q2	<18	6 (28.6)	10 (47.6)	5 (23.8)	0 (0.0)	0 (0.0)	**<**.**001**	13 (61.9)	6 (28.6)	1 (4.8)	0 (0.0)	1 (4.8)	**<**.**001**
	18–25	24 (31.2)	29 (37.7)	17 (22.1)	7 (9.1)	0 (0.0)		42 (54.5)	24 (31.2)	8 (10.4)	2 (2.6)	1 (1.3)	
	26–35	18 (16.4)	33 (30.0)	39 (35.5)	18 (16.4)	2 (1.8)		61 (55.5)	32 (29.1)	13 (11.8)	3 (2.7)	1 (0.9)	
	36–45	34 (29.1)	31 (26.5)	25 (21.4)	23 (19.7)	4 (3.4)		66 (56.4)	33 (28.2)	14 (12.0)	2 (1.7)	2 (1.7)	
	>45	32 (35.6)	36 (40.0)	10 (11.1)	3 (3.3)	9 (10.0)		46 (51.1)	28 (31.1)	11 (12.2)	5 (5.6)	0 (0.0)	
Q3	<18	4 (19.0)	4 (19.0)	6 (28.6)	2 (9.5)	5 (23.8)	.**001**	8 (38.1)	8 (38.1)	3 (14.3)	1 (4.8)	1 (4.8)	**<**.**001**
	18–25	8 (10.4)	23 (29.9)	20 (26.0)	19 (24.7)	7 (9.1)		33 (42.9)	28 (36.4)	11 (14.3)	2 (2.6)	3 (3.9)	
	26–35	9 (8.2)	32 (29.1)	29 (26.4)	34 (30.9)	6 (5.5)		45 (40.9)	39 (35.5)	15 (13.6)	5 (4.5)	6 (5.5)	
	36–45	15 (12.8)	35 (29.9)	32 (27.4)	19 (16.2)	16 (13.7)		49 (41.9)	41 (35.0)	16 (13.7)	5 (4.3)	6 (5.1)	
	>45	18 (20.0)	30 (33.3)	9 (10.0)	13 (14.4)	20 (22.2)		34 (37.8)	33 (36.7)	13 (14.4)	4 (4.4)	6 (6.7)	
Q4	<18	5 (23.8)	9 (42.9)	5 (23.8)	1 (4.8)	1 (4.8)	**<**.**001**	12 (57.1)	6 (28.6)	2 (9.5)	0 (0.0)	1 (4.8)	**<**.**001**
	18–25	31 (40.3)	21 (27.3)	15 (19.5)	9 (11.7)	1 (1.3)		41 (53.2)	24 (31.2)	9 (11.7)	1 (1.3)	2 (2.6)	
	26–35	18 (16.4)	45 (40.9)	26 (23.6)	19 (17.3)	2 (1.8)		60 (54.5)	33 (30.0)	11 (10.0)	3 (2.7)	3 (2.7)	
	36–45	28 (23.9)	45 (38.5)	19 (16.2)	18 (15.4)	7 (6.0)		65 (55.6)	34 (29.1)	12 (10.3)	3 (2.6)	3 (2.6)	
	>45	25 (27.8)	36 (40.0)	7 (7.8)	10 (11.1)	12 (13.3)		48 (53.3)	28 (31.1)	11 (12.2)	4 (4.4)	-	
Q5	<18	0 (0.0)	3 (14.3)	9 (42.9)	1 (4.8)	8 (38.1)	**<**.**001**	9 (42.9)	7 (33.3)	2 (9.5)	1 (4.8)	2 (9.5)	**<**.**001**
	18–25	15 (19.5)	18 (23.4)	15 (19.5)	17 (22.1)	12 (15.6)		34 (44.2)	26 (33.8)	8 (10.4)	4 (5.2)	5 (6.5)	
	26–35	10 (9.1)	29 (26.4)	31 (28.2)	26 (23.6)	14 (12.7)		49 (44.5)	37 (33.6)	11 (10.0)	6 (5.5)	7 (6.4)	
	36–45	21 (17.9)	24 (20.5)	24 (20.5)	26 (22.2)	22 (18.8)		53 (45.3)	38 (32.5)	12 (10.3)	7 (6.0)	7 (6.0)	
	>45	10 (11.1)	11 (12.2)	9 (10.0)	9 (10.0)	51 (56.7)		42 (46.7)	29 (32.2)	10 (11.1)	8 (8.9)	1 (1.1)	

Bold values indicate statistically significant differences (*p* < 0.05). A, Not anxious; B, Slightly anxious; C, Moderately anxious; D, Very anxious; E, Extremely anxious. *p*-values represent comparisons across groups using the chi-square test.

### Association between Pre- and post-intervention anxiety scores

Pre- and post-intervention anxiety levels for waiting room anxiety (Q2), tooth drilling (Q3), scaling and polishing (Q4), and anticipation of dental appointments (Q1) showed moderately positive relationships using Spearman's rank correlation analysis (Table 6) (*ρ* = 0.378–0.491, *p* < 0.001). Anxiety over receiving a local anesthetic injection (Q5) showed a smaller but statistically significant connection (*ρ* = 0.135, *p* = 0.006). These associations show that after interacting with a chatbot, individuals' anxiety levels generally decreased, even if those with higher baseline anxiety tended to stay comparatively more nervous than others.

**Table 6:** Spearman's correlation analysis between Pre- and post-intervention measures of the severity of dental anxiety following chatbot Use.

Pre- Post -intervention Variable	Spearman's *ρ*	*p*-value
Q1. If you were to go to the dentist tomorrow for treatment, how would you feel?	0.418	<.001
Q2. If you were sitting in the waiting room (waiting for treatment), how would you feel?	0.491	<.001
Q3. If you were about to have one of your teeth drilled (using the drill), how would you feel?	0.378	<.001
Q4. If you were about to have your teeth cleaned and polished (removal of tartar and stains), how would you feel?	0.410	<.001
Q5. If you were about to receive a local anesthetic injection in the gum above one of the upper back teeth, how would you feel?	0.135	.006

Values represent Spearman's rho (*ρ*), two-tailed.

## Discussion

This quasi-experimental study discovered that an AI-based chatbot was linked to short-term decreases in anticipatory dental anxiety in five typical situations, such as waiting for appointments, getting injections, scaling teeth, and drilling teeth. By enhancing familiarity and decreasing ambiguity, the chatbot's organized, scenario-specific information, shared worries, and coping strategies probably helped people feel less anxious. However, the absence of a control group precludes drawing inferences about causality, and the effects are probably situational rather than long-term therapeutic advantages. To supplement current anxiety management techniques, the chatbot functions as a low-intensity, pre-treatment informative tool, in contrast to formal psychological or pharmaceutical interventions. These results are consistent with earlier studies that shown that reassuring and educating patients lessens anxiety (1, 6, 9). They also align with recent digital intervention research targeting anticipatory dental anxiety. For example, Tellez et al. (30) reported significant anxiety reductions following a structured, online cognitive-behavioural intervention, with effects sustained over longer follow-up periods. While their multi-session cognitive-behavioural intervention -based approach targeted broader and longer-term dental anxiety, the present study extends this literature by demonstrating that a brief, AI-based conversational tool delivered immediately before treatment may reduce short-term anticipatory anxiety. Despite differences in intervention intensity and duration, both studies support the role of addressing uncertainty and perceived lack of control as central mechanisms in alleviating dental anxiety.

Consistent with previous publications (10–12), the greatest severity of dental anxiety at baseline in our study was associated with dental procedures commonly perceived as intrusive or unpleasant, such as tooth drilling and local anaesthetic injections. Notably, these procedures also demonstrated the largest absolute reductions in anticipatory anxiety following chatbot use, suggesting that structured, procedure-specific information may help reduce uncertainty when patients are confronted with fear-provoking interventions. This is consistent with theoretical models that suggest dental anxiety is primarily caused by uncertainty and perceived lack of control, and that resolving these aspects can significantly reduce suffering (6, 18).

The chatbot's efficacy across all five dental scenarios highlights its ability to alleviate situational worry and anticipatory anxiety that arise prior to treatment. It has been demonstrated that irregular dental attendance and appointment avoidance are significantly predicted by anticipatory anxiety (5, 15). The chatbot may be able to break this avoidance loop and improve long-term oral health habits by intervening before treatment. Pre-appointment digital therapies have been shown to reduce anxiety and increase patient readiness in medical and mental health contexts, supporting their applicability outside dentistry (20, 25, 31).

Gender-based analyses showed that female patients tended to exhibit greater baseline severity of dental anxiety, which is consistent with epidemiological studies reporting a higher prevalence of severe dental anxiety among women (12, 14, 16). Crucially, the AI-powered chatbot significantly reduced anxiety in both men and women, with gender disparities significantly lessened after the intervention. This implies that by offering standardised, nonjudgmental, and patient-centred information, chatbot-based therapy may aid in reducing current anxiety inequities. The chatbot's effectiveness did not seem to be limited by the lack of gender-specific customisation, suggesting that the essential elements of comfort, clarification, and coping advice are generally relevant across genders.

Patients with lower educational attainment also tended to demonstrate greater moderate-to-severe dental anxiety, particularly for invasive procedures. This result is in line with other research that links increased dental anxiety to worse health literacy and a lack of procedural understanding (2, 8, 17). However, after interacting with the chatbot, anxiety decreased significantly and consistently across all educational groups. Interestingly, people with no formal education or only a certificate saw very noticeable benefits. This demonstrates how the chatbot may close knowledge gaps and promote health equity by providing clear, understandable explanations to patients of all educational backgrounds. AI chatbots can improve understanding and reduce anxiety among populations with varying literacy levels, according to earlier research in digital health (22, 32).

The effectiveness of the chatbot intervention was unaffected by socioeconomic position, as indicated by monthly income. All income groups had moderate-to-extreme anxiety at baseline; however, post-intervention decreases were substantial and consistent. Given that lower-income patients frequently encounter obstacles to conventional anxiety treatment techniques, such as behavioural therapy or pharmaceutical sedation, due to cost or restricted access, this conclusion is particularly pertinent (18). AI-based chatbots complement calls for more egalitarian digital health advances in dentistry by providing a scalable, low-cost solution that can be implemented extensively without adding to the clinical load (19, 26).

Age-based analysis provided additional evidence of the intervention's broad applicability. All age groups—including older patients—saw notable decreases in anxiety after using chatbots, while younger and middle-aged individuals reported higher baseline anxiety for drilling and injections. This refutes the notion that older persons may find digital technologies less useful or acceptable. A growing body of research indicates that when user interfaces are intuitive and information is pertinent, well-designed conversational bots may be effectively embraced by people of all ages (31, 33). The results of this study support the viability of using chatbot-based anxiety therapy throughout the adult lifetime.

For most dental scenarios, the correlation analysis between pre- and post-intervention measures showed modest positive associations, indicating that individuals with greater baseline severity of dental anxiety tended to remain comparatively more apprehensive than others even after the intervention. However, anxiety was much lower in all baseline profiles. The deeply rooted “needle-related dread”, which has been demonstrated to be quite resistant to brief educational strategies, may account for the lower connection seen for local anaesthetic injection anxiety (9, 18). The comparatively weaker correlation observed for injection-related anxiety is consistent with literature on needle phobia and blood-injection-injury fear, which suggests that injection anxiety often involves specific phobic components such as conditioned fear, disgust sensitivity, and potential vasovagal responses. These mechanisms may be less responsive to brief informational or conversational interventions alone. Consequently, while chatbot-based support may help reduce uncertainty around injections, patients with marked needle anxiety may require additional behavioural or psychological strategies (e.g., graded exposure, applied tension techniques, or targeted coping skills) to achieve more substantial reductions in anxiety.

While chatbots can generally help people feel less anxious, multimodal methods that integrate digital technologies with behavioral or pharmaceutical techniques may be more beneficial for people with severe procedural phobias. According to our findings, AI-based chatbots can be used in routine dental processes as comforting and educational tools prior to treatments. Traditional chairside anxiety management techniques can be supplemented rather than replaced by these technologies, which are available prior to visits, need little staff participation, and can deliver consistent information at scale. According to earlier research, chatbots can improve patient involvement and lessen psychological pain in settings including managing ongoing medical conditions, mental health, and cancer treatment (22, 25, 34, 35). These kinds of technologies might assist patients in dentistry be ready for treatments, collaborate better, and even have better treatment experiences.

This study has several limitations. Although reductions in anticipatory dental anxiety were observed across demographic groups, the quasi-experimental design without a control group means these changes cannot be attributed solely to the chatbot; alternative explanations such as attention placebo effects, habituation to the clinical environment, or the passage of time may have contributed. The study focused on short-term, self-reported anxiety immediately post-intervention, limiting conclusions about sustained effects, actual dental attendance, treatment compliance, or long-term psychological outcomes. While several mechanisms may plausibly explain how AI chatbots influence anxiety—such as reducing uncertainty through clear, procedure-specific explanations, enhancing perceived control, providing brief emotional support, and improving cognitive processing—these were not directly measured. Self-reported measures may also be influenced by social desirability bias. Additionally, the study did not perform multivariate analyses to adjust for potential confounding variables, which may have influenced the observed changes in dental anxiety. Future randomized controlled trials with appropriate control conditions, longer follow-up, additional psychological and behavioural measures, physiological stress assessments, and exploration of personalised chatbot adaptations are needed to rigorously evaluate causal effects and equity of impact across different patient groups.

## Conclusions

Within the limitations of a pre–post design and a single self-report measure, this study found that interaction with an AI-based chatbot was associated with short-term reductions in anticipatory dental anxiety among adult patients across several common dental scenarios, including more invasive procedures that typically elicit greater fear. Reductions were observed across demographic groups, suggesting that such tools may be broadly acceptable as a low-intensity support. Chatbot-based assistance may complement, but not replace, conventional anxiety management by providing brief, procedure-specific information and helping to reduce uncertainty immediately prior to treatment. However, these findings do not establish causality or long-term effectiveness. Future randomised controlled trials incorporating multiple psychological and behavioural outcomes are needed to evaluate sustained effects, clinical relevance, and potential for personalised applications. Until such evidence is available, AI chatbots should be considered as adjunctive, pre-treatment informational tools that may enhance the patient experience in specific contexts rather than definitive solutions for dental anxiety.

## Data Availability

The datasets presented in this study can be found in online repositories. The names of the repository/repositories and accession number(s) can be found in the article/Supplementary Material.
